# Emerging Roles of Urine-Derived Components for the Management of Bladder Cancer: One Man’s Trash Is Another Man’s Treasure

**DOI:** 10.3390/cancers13030422

**Published:** 2021-01-23

**Authors:** Sarah Minkler, Fabrice Lucien, Michael J. Kimber, Dipak K. Sahoo, Agnes Bourgois-Mochel, Margaret Musser, Chad Johannes, Igor Frank, John Cheville, Karin Allenspach, Jonathan P. Mochel

**Affiliations:** 1 SMART Translational Medicine, Department of Biomedical Sciences, College of Veterinary Medicine, Iowa State University, Ames, IA 50011-1250, USA; sminkler@iastate.edu (S.M.); dsahoo@iastate.edu (D.K.S.); allek@iastate.edu (K.A.); 2 Department of Biomedical Sciences, College of Veterinary Medicine, Iowa State University, Ames, IA 50011-1250, USA; michaelk@iastate.edu; 3 Department of Urology, Mayo Clinic, Rochester, MN 55902, USA; Lucien-Matteoni.Fabrice@mayo.edu (F.L.); Frank.Igor@mayo.edu (I.F.); Cheville.John@mayo.edu (J.C.); 4 Department of Veterinary Clinical Sciences, College of Veterinary Medicine, Iowa State University, Ames, IA 50011-1250, USA; abmochel@iastate.edu (A.B.-M.); mmusser@iastate.edu (M.M.); cmj15@iastate.edu (C.J.); 5 SMART Pharmacology, Department of Biomedical Sciences, College of Veterinary Medicine, Iowa State University, Ames, IA 50011-1250, USA

**Keywords:** bladder cancer, organoids, exosomes, precision medicine, one health

## Abstract

**Simple Summary:**

Urinary bladder cancer (UBC) is one of the most common and deadly cancers worldwide, with many patients not responding to chemotherapy, or presenting with serious adverse effects after chemotherapy. Yet, current bench side assays provide limited accuracy for predicting therapeutic response to chemotherapeutic drugs. The aim of this review is to demonstrate the potential of urinary-derived extracellular vesicles and UBC-organoids to serve as predictive biomarkers for this cancer. Specifically, molecular subtyping of urine-derived extracellular vesicles has the potential to provide insights into the molecular stratification of the tumor, while urinary organoids will allow for individualized chemotherapy testing in the context of precision medicine.

**Abstract:**

Urinary bladder cancer (UBC) is the most common malignancy of the urinary tract in humans, with an estimated global prevalence of 1.1 million cases over 5 years. Because of its high rates of recurrence and resistance to chemotherapy, UBC is one of the most expensive cancers to treat, resulting in significant health care costs. The development of innovative molecular and cellular tools is necessary to refine patient stratification and help predict response to treatment. Urine is an underused resource of biological components shed from bladder tumors, such as exfoliated cells and extracellular vesicles, that could serve as molecular fingerprints and provide valuable biological insights into tumor phenotype and mechanisms of resistance to chemotherapy. Additionally, characterization of urine-derived extracellular vesicles and cells could be used as reliable biomarkers for prediction of response to neoadjuvant therapy.

## 1. Introduction

Urinary bladder cancer (UBC) is a common urogenital malignancy causing approximately 80,000 new cases and 18,000 deaths each year in the United States alone [1,2]. Urothelial carcinoma accounts for 90% of bladder cancers and can be categorized into non-muscle invasive bladder cancer (NMIBC) and muscle-invasive bladder cancer (MIBC) subtypes; although the majority of UBC present as NMIBC, the MIBC subtype is associated with the highest risk of developing metastases. Overall, 75% of patients diagnosed with high-risk bladder cancer will experience tumor recurrence, advancement of cancer, or decease within 10 years of their diagnosis [1]. Transurethral resection (TUR) of all visible lesions is a standard treatment for NMIBC but is associated with a high recurrence rate [3]. Intravesical chemotherapy and immunotherapy have demonstrated significant benefit in delaying disease recurrence in patients with NMIBC [4]. In MIBC patients, neoadjuvant chemotherapy with platinum-based drugs has been offered prior to local definitive treatment and has been associated with lower rates of recurrence and survival benefits [5,6]. Recently, a myriad of clinical trials has been launched to investigate the efficacy of immune checkpoint inhibitors combined with neoadjuvant therapy [7,8,9]. The outcome of these clinical trials may significantly change the therapeutic landscape of MIBC patients as half of MIBC patients are not eligible to receive platinum-based neoadjuvant chemotherapy [10]. In patients receiving treatment with neoadjuvant therapy, pathological complete response (pCR, pT0N0) rates have been observed in 20% to 50% of cases [6,7,9]. While there is still room to develop more effective neoadjuvant therapies and increase pCR rates, avoiding surgery in bladder cancer patients who completely respond to neoadjuvant therapy is a continuing challenge faced by many urologic oncologists. Additionally, disease recurrence has been reported in a subset of patients initially diagnosed with pCR, highlighting the need to identify patients who present with occult metastasis at the time of surgery, as they could benefit from active surveillance and additional therapy to prevent disease recurrence. There is a critical need to identify those patients who can safely avoid surgery following neoadjuvant therapy, as well as those who need follow-up and additional therapy [11,12,13]. In this review, we will discuss several emerging platforms that have strong potential to address these needs. First, we will describe challenges and clinical opportunities of ex vivo patient-derived tumor systems including urine-derived tumor organoids as a preclinical drug testing platform for patients diagnosed with bladder cancer. Second, we will provide an overview of urine-based liquid biopsies, in particular with tumor-derived extracellular vesicles that can help monitoring response to treatment and identify complete responders.

## 2. Current Precision Medicine-Approaches for the Treatment of Bladder Cancer Are Promising but Have Significant Drawbacks

To date, bladder cancer management decisions have been based on conventional histological features including tumor stage, lymph node status, and histology variant at the time of diagnosis. Half of patients treated with neoadjuvant therapy, however, do not respond to treatment, highlighting our current inability to accurately predict those patients who will respond to chemotherapy [6,7,9]. Pathological factors have been evaluated for their predictive value in the context of muscle-invasive bladder cancer [14]. Specifically, patients with pure urothelial carcinoma have ~11 times more chance to experience pathological complete response post-neoadjuvant therapy (NAT) compared to tumors with histological variants or mixed tumors. While pure urothelial carcinoma constitutes ~70% of cases of bladder cancer, the remaining cases are histologic variants or have mixed histological features [15]. This intratumor heterogeneity is a significant hurdle to any clinical decision-making involving best choice of treatment for patients with UBC [16].

Recent technological advances have allowed for efficient deep molecular profiling of bladder cancer tumors to support prediction of clinical outcomes and responses to therapy [17,18,19]. Transcriptomic profiling of biopsy and cystectomy specimens has, for instance, revealed distinct molecular subtypes of bladder cancer [18,20,21,22,23]. Similar to histology, molecular classification reveals important tumor heterogeneity with co-existence of luminal and basal subtypes within the same tumor in ~30% of cases [24,25]. However, while studies agree on gene expression signatures that identify each molecular subtype, they have shown conflicting results with regards to prediction of response to chemotherapy. Two recent studies, including one meta-analysis of 16 transcriptomic datasets, showed no significant difference in response rates to chemotherapy between tumor subtypes [20,21]. Overall, these findings collectively support the fact that UBC is a multifactorial disease whose genomic, transcriptomic, and epigenomic diversity represent a significant challenge in treatment decision-making. Additionally, the high cost of such molecular analyses and the relatively long turn-around time for data collection and downstream bioinformatic interrogation are further obstacles for personalized medicine applications [20,21]. These limitations underscore the need to develop additional biological resources that can improve patient stratification and better predict response to chemotherapy.

## 3. Preclinical 2D and Patient-Derived Xenograft Models Bring Value to Drug Discovery but Have Limited Bedside Applications

The increasingly recognized complexity and heterogeneity of bladder cancer has posed a major challenge to predicting treatment response. New tumor models generated from patient’s tumor specimens, such as primary cell lines and patient-derived xenografts, have gained attention for preclinical drug testing. Conventional two-dimensional (2D) culture of urothelial carcinoma (UC) cells [26] has traditionally been used for prediction of chemotherapeutic efficacy [27]. Over the years, a large number of bladder cancer cell lines has been established, recapitulating genomic and phenotypic heterogeneity of bladder cancer [28]. However, the majority of bladder cancer cell lines have been established from muscle-invasive and metastatic bladder cancer. While non-muscle invasive bladder cancer is the most common form of urothelial carcinoma, establishment of primary cell lines has not been successful [28,29]. Although 2D cell lines can expand rapidly and offer the possibility for high-throughput drug screening, they do not faithfully reproduce the 3-dimensional nature and cellular diversity of native bladder cancer. Compounding this, cancer-derived 2D cell lines typically exhibit genetic drift after multiple passages [30]. These limiting factors likely contribute to failure in predicting in vivo drug response in cancer patients using only 2D cell lines. 

Patient-derived xenografts (PDX) is an approach whereby patient tumor fragments are implanted into immunocompromised mice to generate tumors that recapitulate genomic and phenotypical features of a patient’s original tumor [31,32]. PDX have value in both better understanding tumor biology and evaluating the efficacy of FDA-approved anticancer therapies or novel targeted treatments [33]. Although PDX models present an exciting opportunity for improving predictive value of preclinical studies, there are several hurdles to their translation into the clinic. The lack of an immune system in the immunocompromised host makes PDX models inadequate for modeling immune response and testing immunotherapies. Further, engraftment rates tend to positively correlate with tumor grade, meaning that low-grade patient tumors may not lead to a high yield of viable mouse tumors [34,35]. Finally, engrafted tumors can take several months to grow. This is a critical drawback for their application in translational medicine as, in the neoadjuvant setting, treatment is usually initiated within 3-4 weeks from the time of diagnosis. An ideal tumor model would combine the rapid growth and high-throughput potential of 2D models with the faithful recapitulation of host tumor microenvironment provided by PDX platforms.

## 4. Patient-Derived Tumor Organoids: a Preclinical Platform for Individualized Prediction of Drug Response

Patient-derived tumor organoids represent a novel and superior model to identify and evaluate the efficacy of anticancer drugs. Tumor organoids are ex vivo mini tumors grown from a patient’s tumor fragments. By maintaining the original cellular composition of tumors, tumor organoids better reflect the physiology of tumor growth compared to conventional models such as two-dimensional primary cell lines [36]. Therefore, UBC-derived organoids have the potential to provide an ex vivo model of bladder cancer that can functionally predict treatment responses [36]. Moreover, many of the strategies outlined above rely on the invasive collection of large tissue specimens through cystectomy. We propose that culture of patient-derived organoids for chemo-sensitivity drug screening be performed on non-invasively obtained urine samples, as previously described in dogs [37]. This constitutes a significant innovation and advantage over currently established methods in the context of precision medicine. 

Organoids can be propagated from bladder cancer cells derived from urine [36,37,38,39] or bladder biopsies [40]. Urine and biopsy-derived organoids have been shown to recapitulate molecular subtypes and heterogeneity [36,37,38,39]. Specifically, steady expression of urothelial cell markers (e.g., CK7, CK20, UPK3A, and CD44) has been reported on the luminal side of UBC organoids along with that of the proliferation marker Ki67 [36,40]. In addition, xenografted urine-derived organoids were able to cause tumorigenesis in immunodeficient mice, demonstrating their ability to maintain oncogenic properties ex vivo. Characterization of organoids can be done through an array of cellular and molecular techniques including immunohistochemistry, RNA-Seq, proteomics, and others [33,41]. Current research using organoids to study cancer biology offers promising preliminary results. Melanoma-derived organoids, for example, have shown to be responsive to immune checkpoint inhibitors such as PD-1 and CTLA-1 antibodies [42,43]. This is a significant advantage over other model systems because we can now study the interaction of immune and tumor cells, through their co-culture, to refine prediction of drug response ex vivo. In addition, organoids have been shown to provide detailed information on the tumor microenvironment and stroma [44]. While organoids offer a promising alternative to the 2-D and xenografted models that are mentioned above, they do have drawbacks themselves. Organoids require skill to grow and cultivate and depending on the sample, can have small or no yields [45], making them a difficult tool for some researchers to use. After isolation, growth in a basement membrane or Matrigel matrix could have potential limitations on drug diffusion into the organoids themselves [45], hindering their ability to accurately predict drug response. As further utilization of organoids in research continues to expand, these issues will have to be addressed for their use in translational medicine.

## 5. Dogs with Bladder Cancer Are a Highly Relevant Model for MIBC

While murine models do have their place in the study of bladder cancer, they typically do not reflect the biological behavior of MIBC in human patients [46]. First, the urothelium of mice is inherently refractory to developing cancer and tumors typically do not metastasize in mice as they do in humans [46,47]. Second, genetically modified mouse models do not effectively mimic the heterogeneity of the human patient population. The MBT-2 model is frequently used for the study of orthotopic bladder cancer, but intravesical drug studies cannot be performed due to the death of the mouse from large tumor growth [48]. In a study by Seo et al., lentiviruses were used to transfect MBT-2 cells, transplanted in immunocompetent mice, with a RNA silencer that targeted the c-myc oncogene and proved to be effective in preventing tumor growth [48]. This new utilization of MBT-2 cells could allow for its application in the study of intravesical drug trials for UBC treatment. Another popular model used in the study of bladder cancer is the F344-AY27 rat model. This model can effectively be used to study drug treatment potency [49], but as mentioned above, this model does not emulate human tumor growth and genetic composition [47]. Even human xenograft models are not ideal for all purposes, as the tumors are transplanted into immunocompromised animals [47]. Dogs, on the other hand, are a well-recognized, natural disease model of human MIBC, with very similar molecular features, tumor heterogeneity and subtypes, metastatic behavior, as well as treatment responses [50,51,52,53]. Some markers associated with MIBC and poor prognosis in human bladder cancer patients (Keratin 7 and CD44) were upregulated in canine organoids, consistent with the finding that dogs present most commonly with MIBC [36,52,54]. 

Research groups are currently investigating the value of using canine bladder cancer organoids for the prediction of drug response and combination [36]. Cell viability assays can determine the lethal drug combination needed to effectively treat for UBC. Herein, we propose to use a spontaneously occurring, analogous disease which constitutes a highly relevant model for UBC in people. Noteworthily, the FDA requires preclinical safety and efficacy data from rodent and non-rodent animal models (commonly dogs) prior to testing of novel drug candidates in human clinical trials. Therefore, we propose that future therapeutic leads for UBC be screened ex vivo using canine organoids to select the most promising drug candidates. Subsequently, these novel therapeutics could be tested in vivo in dogs with bladder cancer prior to formal clinical testing in human patients with UBC. The establishment of canine organoids as an ex vivo model in combination with the ability to test new candidate drugs in preclinical trials in dogs may therefore represent a quantum leap in comparative oncology for the faster development of viable treatment options for MIBC. Importantly, while dogs are excellent for modeling disease phenotypes in humans, access to human UBC organoids would allow for greater research and testing into drug resistance of human UBC subtypes.

## 6. Tumor-Derived Extracellular Vesicles for the Monitoring of Bladder Cancer Treatment Response

Monitoring response to chemotherapy and identifying complete responders are two common challenges in urology oncologist daily practice. The close contact with the urothelium makes urine an attractive approach to detect the presence of exfoliated tumor cells and tumor derivatives (including soluble proteins and other factors with diagnostic potential). As such, collection of urine specimens offers distinct advantages over tissue biopsies due to the non-invasive nature of the method and the ability to perform longitudinal sample collection during the 6-to-8-week course of NAT administration. Extracellular vesicles (EVs) are one tumor derivative with recognized emerging diagnostic potential [55]. EVs are nano-scale (<1000 nm) membrane-bound structures released by all living cell types, including tumor cells [56]. EVs contain diverse molecule cargo (extracellular DNA, RNA, lipids, and proteins) and surface molecules reflecting their parental cells and can be isolated from an array of biofluids including urine [57,58]. Originally characterized as professional “garbage bags” carrying waste cellular products [59], subsequent research has shown that EVs are key facilitators of intercellular communication by mediating cargo transfer between cells [60,61]. As EVs can be found in relative abundance, their enumeration in biofluids offers quantitative advantages over the paucity and short half-life of other tumor biomarkers such as circulating tumor cells (CTCs) and circulating tumor DNA (ctDNA) [62]. Since RNA and DNA are packaged within EVs and protected from degradation by a phospholipid bilayer, their analysis may provide additional diagnostic and prognostic value, and prove useful for monitoring of treatment response [63]. In addition, EVs are shed from metabolically active cells which provide a more accurate reflection of tumor burden, while ctDNA is derived from apoptotic cells [64]. As of October 1st 2020, 24,002 publications with the search key “extracellular vesicles” have been indexed on PubMed, with 75% of these publications being released within the last seven years. Strikingly, more than 50 biotechnology companies focusing on diagnostic and therapeutic applications of EVs have emerged in the last decade. These numbers reflect the translational potential of EVs for clinical application in biomedical sciences.

## 7. Molecular Composition of Urine-Derived EVs in Bladder Cancer Patients

A rich body of literature has begun to describe the molecular composition of urine-derived EVs in bladder cancer patients vs. healthy individuals [65]. These studies show that RNA (also referred to as extracellular RNA or exosomal RNA) is one of the most abundant molecules found in EVs. Among RNA subclasses, miRNA and ribosomal RNA represent more than 80% of total RNA composition of EVs [66]. Specifically, miRNAs from the miRNA-200 (miR-141-3p/5p, miR-200a/b/c-3p/5, and miR-205-3p/5p) family have been isolated from urine exosomes [67]. These miRNAs are associated with epithelial-to-mesenchymal transition (EMT) and reflect highly invasive tumor cell types due to loss of epithelial proteins leading to reduced cell adhesion [68]. Several other miRNAs have shown promise as potential biomarkers. For instance, miR-375 was found in EVs of patients with high-grade bladder cancer, while miR-146a was found in EVs of patients with low-grade tumors [69]. The long non-coding RNA (lncRNA) HOTAIR (HOX transcript antisense RNA) has also been found in urinary exosomes from patients with UBC [70]. HOTAIR is known to expedite tumor initiation and assist in tumor progression in many different cancers [71,72,73,74,75]. Specifically, Berronodo et al. (2016) showed that knocking out HOTAIR in UBC cell lines resulted in reduced cell migration and invasion, demonstrating a potential therapeutic use in UBC [70]. Other lncRNAs found in urinary EVs include LINC00355, UCA1-203, and MALAT1. Interestingly, all three of these lncRNAs have significantly higher expression in UBC-derived vesicles compared to healthy controls [76]. Exosomal DNA isolated from urine-derived EVs could be another potential biomarker for UBC. Indeed, using deep sequencing, exosomal DNA was found to have somatic mutations that are commonly found in UBC cells and provide insights into the genetic abnormalities of UBC tumors [77]. Table 1 presents a summary of RNA biomarkers found in urine derived extracellular vesicles.

In addition to RNA, exosomes also have an extensive protein complement that reflects both the cargo of the vesicles and structural proteins that are embedded in the membrane of the EVs. All EV proteins are derived from the parental cell and as such, proteins enriched in EVs secreted by UBC tumors have strong theoretical diagnostic and prognostic potential. A number of studies have profiled the proteomes of EVs isolated from urine of bladder cancer patients and identified potential EV protein markers of UBC [78,79]. Differences in EV isolation techniques and mass spectrometry pipelines manifest in limited consensus, but several of these studies are noteworthy. Smalley et al. (2008) identified nine EV proteins differentially expressed in UBC patients. Most of these proteins are membrane-associated, which may facilitate diagnostic methods that recognize EV surface proteins, and two, Mucin 4 and NRas, had previously been proposed as biomarkers in other forms of cancer [80]. More recently, Lee et al. identified 56 urinary EV proteins upregulated in UBC samples and advanced four for further validation: Mucin 1, a glycoprotein involved in the pathogenesis of several cancers; carcinoembryonic antigen (CEA), a glycoprotein associated with angiogenesis and immune evasion; epidermal growth factor receptor kinase substrate 8-like protein 2 (EPS8L2), associated with endometrial cancer; and Moesin, an ERM-family protein associated with metastasis [81]. Most comprehensively, Chen et al. (2012) examined EV proteomes from aged-matched and graded UBC patients [82]. They identified 107 potential biomarkers but speculated many may be derived from blood in the urine of UBC patients and not reflective of bladder tissue. Tumor Associated Calcium Signal Transducer 2 (TACSTD2) was predicted as a UBC specific biomarker and when tested in 140 UBC patients, was diagnostic in 74% [82,83]. This underscores the potential for EV proteins as UBC biomarkers but also emphasizes that extensive validation of novel EV biomarkers is needed and might suggest that multiplexing several protein targets (or indeed modalities; miRNA and proteins) may improve specificity and accuracy [83]. See Table 1 for a summary of identified protein biomarkers in urine-derived extracellular vesicles.

## 8. Opportunities for Translating Research on Extracellular Vesicles from Bench to Bedside

One of the major limitations of previous studies focusing on the molecular characterization of UBC-derived EVs lies in the use of total urinary EVs for RNA and proteomic profiling. Urinary EVs may originate from non-malignant cells of the urinary tract including the prostate, kidneys, and the upper urinary tract [84]. Therefore, it is important to discriminate UBC-derived EVs from the heterogeneous population of urinary non-tumor-derived EVs, including those produced by the healthy urothelium. Reliable isolation of UBC-derived EVs is dependent on recognizing markers exclusively and consistently expressed on the surface of EVs released by tumor cells. These cell-surface markers can be utilized to collect UBC-derived EVs through magnetic bead-based immunocapture or fluorescence-activated particle sorting across multiple platforms [85,86]. In the absence of tumor-specific antigens, isolation of bladder-specific EVs (from both tumors and normal cells) can be acceptable as a way to enrich the diagnostic EV pool with UBC-derived EVs and to minimize confounding urinary EVs with other cellular origins. 

As an example of these strategies, uroplakins are attractive candidates for isolating bladder cancer-derived EVs. Uroplakins are a family of four highly glycosylated cell-surface proteins (1A, 1B, 2, 3) involved in urothelium plaque formation and permeability [87]. Uroplakins are not expressed by non-urothelial tissue, have limited expression in normal urothelium but high expression in bladder cancer [88,89]. Uroplakin 2 is routinely used for immunohistochemical diagnostic of urothelial carcinoma, with more than 80% of patients having detectable levels of uroplakins [90,91], and uroplakins have been found in urinary EVs isolated from patients with bladder cancer [84]. These proteins may represent suitable markers by which it is possible to isolate urinary EVs specifically released by the urothelium and bladder cancer tumor cells. 

One of the major challenges in the development of EV-based liquid biopsy strategies has been the lack of reliable and standardized isolation and detection techniques [92]. EV isolation typically requires time-consuming and labor-intensive methods centered on differential ultracentrifugation protocols or kits that employ size-exclusion or affinity columns that are not convenient or practical in the clinical setting. The challenges to clinically validating a diagnostic or prognostic assay based on EVs that meets preferred performance characteristics are acknowledged, but success is achievable [92]. Additionally, while no such assay has been developed for bladder cancer yet, recent advances have been made for other tumor types such as prostate cancer. The ExoDx Prostate IntelliScore (EPI) from Exosome Diagnostics (a Bio-Techne brand) is a urine-based test that combines ultrafiltration-based bulk EV isolation and RT-qPCR to measure expression of three genes highly upregulated in prostate cancer: PCA3 (prostate cancer antigen 3), ERG (V-ets) erythroblastosis virus E26 oncogene homologs), and SPDEF. A risk score (0–100) is calculated based on gene expression profiles that can predict the presence of clinically-significant prostate cancers (Grade Group ≥ 2) [93,94]. Following two successful clinical studies, the EPI test has been included in the NCCN guidelines for early detection in men for both initial and repeat biopsy. Another EV-based assay, the ClarityDx from Nanostics, is currently investigated to refine prostate cancer risk stratification [95]. Unlike the EPI test, the ClarityDx relies on direct enumeration of circulating prostate cancer-derived EVs from a simple blood draw using microflow cytometry [96,97]. Prostate cancer-derived EVs are characterized as positive for the following three markers: (1) PSMA (prostate-specific membrane antigen), (2) polysialic acid (PolySia), and (3) ghrelin receptor (GHSR). Flow cytometry quantification of EVs positive for these markers combined with machine learning-assisted data analysis provides a diagnostic accuracy of 0.81 (AUC) with 95% sensitivity and 97% negative predictive value for Grade Group ≥ 3 prostate cancer. Microflow cytometry is a state-of-the-art technology allowing for multiparametric phenotyping and enumeration of EVs at an unprecedented resolution. More importantly, it does not require any isolation/purification step and rapid enumeration of EVs from a very small volume of sample provides an excellent opportunity for characterization and quantification of EVs in body fluids. Despite the need for standardization and validation, this technology holds a lot of promise for clinical studies and it provides great potential for prediction of therapy response and improvement of patient outcome [98,99,100,101]. The EPI and the ClarityDx assays rely on distinct biofluid sources and different analytical platforms but they demonstrate the clinical value of EV-based liquid biopsies for the management of cancer patients. Upon prospective studies with large population cohorts, such assays will likely see the light in patients with bladder cancer.

## 9. Conclusions

Urinary bladder cancer, especially in its muscle-invasive form, is associated with an extremely poor survival rate [102,103]. As of today, many of the underlying mechanisms of UBC remain unknown, making it difficult to diagnose early and treat effectively. Additionally, UBC exhibits many heterogeneous subtypes and a broad range of disease phenotypes, such that therapeutic response to conventional chemotherapy is extremely variable among patients. The development of new molecular and cellular tools, such as UBC-derived EVs and organoids, provide an opportunity to streamline the diagnosis and characterization of UBC tumor subtypes. Additionally, 3D tumor organoids have been shown to retain their oncogenic-like properties when cultured ex vivo and could be used as a platform for drug screening purposes prior to clinical evaluation in patients with UBC.

## Figures and Tables

**Table 1:** Table summarizing contents of urine derived extracellular vesicles as potential biomarkers in cancer reserach. (**A**) Summary of extracellular vesicle RNA biomarkers and their functions. (**B**) Summary of extracellular vesicle protein biomarkers and their functions.

Biomarkers	Function/Association
A. RNA	
miR-200	Epithelial-to-mesenchymal transition
miR-375	Associated with high grade bladder cancer
miR-146	Associated with low grade bladder cancer
HOTAIR	Tumor initiation and progression
LINC00335	Cell migration and invasiveness
UCA1-203	Cell proliferation, migration, invasiveness and drug resistance
MALAT1	Cell proliferation and motility and inhibition of apoptosis
B. Proteins	
Mucin-1	Pathogenesis of many cancers
Carcinoembryonic antigen (CEA)	Angiogenesis and immune evasion
Epidermal growth factor receptor kinase 8-like protein	Associated with endometrial cancer and part of the epidermal growth factor receptor pathway
Moesin	Cancer metastasis
Tumor associated calcium signal transducer 2	Cell surface receptor that transduces intracellular calcium
